# What prevents us from reusing medical real-world data in research

**DOI:** 10.1038/s41597-023-02361-2

**Published:** 2023-07-13

**Authors:** Julia Gehrmann, Edit Herczog, Stefan Decker, Oya Beyan

**Affiliations:** 1 University of Cologne, Faculty of Medicine and University Hospital Cologne, Institute for Biomedical Informatics, Cologne, Germany; 2 Vision & Values, Brussels, Belgium; 3 Chair of Computer Science 5, RWTH Aachen University, Aachen, Germany; 4 Department of Data Science and Artificial Intelligence, Fraunhofer FIT, Sankt Augustin, Germany

**Keywords:** Outcomes research, Preclinical research, Biomarkers, Epidemiology, Genetics research

## Abstract

Medical real-world data stored in clinical systems represents a valuable knowledge source for medical research, but its usage is still challenged by various technical and cultural aspects. Analyzing these challenges and suggesting measures for future improvement are crucial to improve the situation. This comment paper represents such an analysis from the perspective of research.

## Introduction

Recent studies show that Medical Data Science (MDS) carries great potential to improve healthcare^1–3^. Thereby, considering data from several medical areas and of different types, i.e. using multimodal data, significantly increases the quality of the research results^4,5^. On the other hand, the inclusion of more features in an MDS analysis means that more medical cases are required to represent the full range of possible feature combinations in a quantity that would be sufficient for a meaningful analysis. Historically, data acquisition in medical research applies prospective data collection, e.g. in clinical studies. However, prospectively collecting the amount of data needed for advanced multimodal data analyses is not feasible for two reasons. Firstly, such a data collection process would cost an enormous amount of money. Secondly, it would take decades to generate enough data for longitudinal analyses, while the results are needed now. A worthwhile alternative is using real-world data (RWD) from clinical systems of e.g. university hospitals. This data is immediately accessible in large quantities, providing full flexibility in the choice of the analyzed research questions^6,7^. However, when compared to prospectively curated data, medical RWD usually lacks quality due to the specificities of medical RWD outlined in section 2. The reduced quality makes its preparation for analysis more challenging. Table 1 summarizes the advantages and disadvantages of both data curation strategies.

**Table 1:** Advantages and disadvantages of prospective data curation and secondary reuse of RWD for MDS.

Aspect	Prospective data curation	Reusing real-world data
Cost	High	Low
Quality	High	Low
Availability	With delay	Immediately
Abundance	Limited by protocol design and cost	Vast amount
Flexibility of the analysis	Limited by protocol design	High
Effort of data preparation	Low	High

Considering all the above-mentioned aspects, secondary use of RWD is a great opportunity to immediately enable comprehensive and meaningful MDS analyses. These, in turn, promise increased clinical process efficiency, higher patient safety, performant clinical decision support systems, personalized care and improved healthcare system sustainability^1^. Yet MDS reusing RWD is still not established in practice for various reasons^2^. One such reason is the lack of standardized data curation frameworks specifying how to access and combine multimodal clinical data from operational clinical systems^8,9^. To maximize the usability of medical RWD for research, such a framework should support data management according to the “FAIR” paradigm, which states that properly managed data should be discoverable, accessible, interoperable, and reusable (FAIR). These are high-level principles, i.e., they do not specify a specific technology, method, or standard, but rather serve as guidance^10^. The extent to which a data set fulfills the four principles is known as its FAIRness. The process of increasing the FAIRness of data is referred to as FAIRification^11^.

To support the scientific reuse of medical RWD with maximal FAIRness, the German Medical Informatics Initiative (MI-I) established Data Integration Centers (DIC) and Medical Data Integration Centers (MeDIC) at German University Hospitals^12–16^. The challenges encountered at MeDIC Cologne have compelled us to write this comment paper, which aims to address key issues surrounding the reuse of medical real-world data (RWD) in research. In addition to the technical challenges extensively discussed in existing literature, we also delve into the cultural aspects and uncertainties that scientists, patients, and governing entities confront when reusing medical RWD. As part of our contribution, we propose high-level measures to enhance the reusability of medical RWD for research purposes. Finally, we evaluate the current usability of medical RWD in terms of the FAIR principles. Our insights draw upon personal experiences, as well as relevant findings from recent English and German literature (2016–2022) obtained through Google Scholar. However, it is important to note that the challenges and measures presented in this paper primarily reflect our personal perspectives and may not encompass all possible aspects.

## Specificities of medical real-world data

The main difference between medical data and other scientific data is its high level of intrinsic sensitivity requiring thorough preservation of privacy^17^. Medical data can contain a variety of information, including demographics, healthcare provider notes, radiological findings, results of laboratory or genetic tests, presence or absence of biomarkers, administrative information, case summaries for clinical registries, biometric information, patient-reported information and recordings from medical devices or wearable sensors^18,19^. This variety is also reflected in the data formats available that range from tabular, time series and natural language data to images and videos^20^. Issues that are typically attributed to the secondary use of medical RWD are their low volume, i.e. small data set sizes, their high sparsity and their tendency towards poor quality^21^. These issues result from the inherent heterogeneity of treatments, outcomes, study design, analytical methods, and approaches for collecting, processing and interpreting data in the medical field^19^. Thus, the availability and quality of features for a patient strongly depend on the conditions present, the treating or examining department, comorbidity as well as current or previous examination results.

## Reusing medical real-world data for medical data science

The main tasks in facilitating, or even enabling, the reuse of medical RWD in a research context are to promote interoperability, harmonization, data quality, and ensure privacy, to optimize the retrieval and management of patient consent, and to establish rules for data use and access^12,13^. These measures aim to address the various challenges of scientifically reusing routine clinical data described below.

### Challenges in balancing benefits and harms

Personal, i.e. non-anonymized medical data, is inherently sensitive^1,17,22^. As a result, uncertainties in MDS project preparation and execution arise for all roles involved in performing research on medical RWD, i.e. for patients, researchers and governing entities. The patients may lack trust in research using their personal data. Concerns about data misuse, becoming completely transparent and data leakage - especially in the case of long-term storage - can result in the patients overprotecting their own data and not giving their consent for its reuse in research^23–25^. On the other hand, it has also been shown that most EU citizens support secondary use of medical data if it serves further common good^24^. So, convincing patients about the social expediency of MDS can decrease their ambivalence and avoid overprotection. This can be achieved, for example, by reporting on MDS success stories^13^. A second important aspect is patient empowerment by informing patients about the processing and use of their data through open scientific communication and enabling their active engagement in the form of a dynamic consent management^12,23^.

However, there are also concerns on the part of the researcher resulting e.g. from a lack of explicit training in a complex landscape of ethical and legal requirements. These could be mitigated by discussions in interdisciplinary team meetings but differences in the daily work routine make it difficult to arrange such meetings^8,9,18,21^. As a consequence of unresolved concerns, researchers could delay or even cancel their MDS projects. Moreover, even governing entities such as data protection officers and ethics committees exhibit a certain level of uncertainty regarding permissible practices in MDS. They tend to overprotect the rights of the patients whose medical data is to be used while underestimating the necessity of reusing medical RWD for research purposes^9,23,26,27^. This leads to restrictive policies hindering scientific progress.

**Fig. 1:**
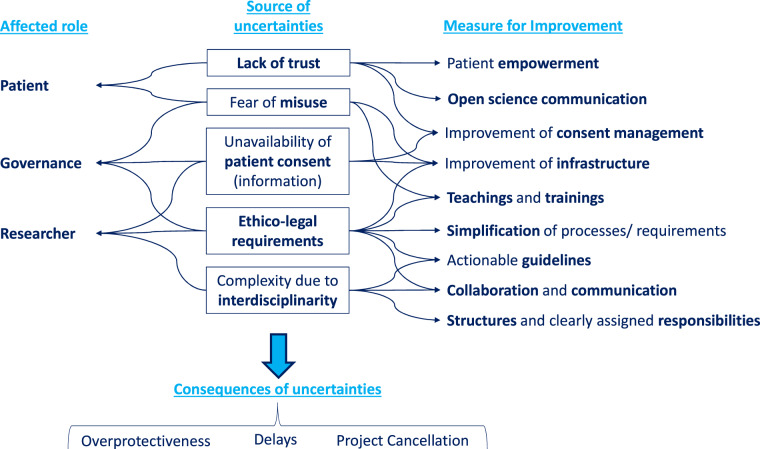
Sources and consequences of uncertainties that lead to significant challenges in the reuse of medical RWD. The sources of uncertainties are individually assigned to the roles they affect and possible measures to counteract them.

In general, education is a promising approach to address the uncertainties mentioned above. Technical training for medical researchers and governing entities as well as ethical and legal training for technical experts can increase confidence in project-related decision making^1,18,23,24,27,28^. The same effect can be achieved by developing MDS guidelines and actionable data protection concepts (DPC)^13–16^. A good example is the DPC of the MI-I that was developed in collaboration with the German working group of medical ethics committees (AK-EK)^12^. Figure 1 summarizes the sources and consequences of the aforementioned uncertainties that lead to significant challenges in the reuse of medical RWD. Each source of uncertainty is associated with the roles it affects and possible measures to mitigate its impact. The challenges posed by these uncertainties are discussed in more detail below.

#### Uncertainties due to the legal framework

As mentioned above, the complex legal landscape resulting from various intervening laws contributes significantly to the uncertainty surrounding the reuse of medical RWD. At the European level, the General Data Protection Regulation (GDPR) holds substantial influence over the legal framework. In general, it prohibits the processing of health-related personal data (GDPR Art. 9 (1)) unless the informed consent of every affected person is given (GDPR Art. 9 (2a)) or a scientific exemption is present (GDPR Art. 9 (2j)). The latter is the case if the processing is in the public interest, secured by data protection measures, and adequately justified by a sufficient scientific goal. However, substantiating the presence of such a scientific exemption poses significant challenges^29,30^. Similarly, or even more difficult, is obtaining informed consent of patients after they have left the clinics. As such, both GDPR-based possibilities to justify the secondary use of RWD in research are difficult to implement in practice^26,29^. If the processing is legally based on the scientific exemption, GDPR Art. 89 further mandates the implementation of appropriate privacy safeguards supported by technical and organizational measures. Additionally, it stipulates that only the data necessary for the project should be utilized (principle of data minimization)^30,31^. This ensures the protection of sensitive personal data, but also introduces further challenges for the researchers.

The situation becomes further complicated due to the GDPR allowing for various interpretations by the data protection laws of EU member states^30,31^. Moreover, there are country-specific regulations, such as job-specific laws, that impact the legal framework of MDS^31^. This complex scenario poses particular challenges for international MDS projects^29^. As a result, identifying the correct legal basis and implementing appropriate data protection measures becomes exceptionally difficult^29,30^. This task, crucial in the preparation of clinical data set compilation, necessitates not only technical and medical expertise but also a comprehensive understanding of legal aspects. Thus, a well-functioning interdisciplinary team or researchers with broad training are essential.

Analyses of the current legal framework for data-driven medical research suggest that this framework is remote from practice and thus inhibits scientific progress^31,32^. To address these limitations, certain legal amendments or substantial infrastructure enhancements are necessary. Particularly, the infrastructure should focus on incorporating components and tools that facilitate semi-automated data integration and data anonymization. Although the current legal framework permits physicians to access, integrate, and anonymize data from their own patients, they often lack the technical expertise and time to effectively carry out these tasks. By implementing an infrastructure that enables semi-automated data integration and anonymization, researchers would be able to legally utilize valuable medical RWD without imposing additional workload on physicians^29,30^. Attaining a fully automated solution is not feasible since effective data integration and anonymization, leading to meaningful data sets, necessitate manual parameter selection by a domain expert. Nonetheless, by prioritizing maximal automation and specifically assigning domain experts to handle the manual steps in the process, rapid and compliant access to medical RWD, along with reduced uncertainties for researchers, can be achieved.

#### Ethical considerations and overprotectiveness

Not only the legal framework, but also ethical considerations can cause uncertainties. These can affect the patients and researchers but, in the context of an MDS project, especially the ethics committees as they have to judge whether a project is ethically justifiable. There are a variety of ethical principles to be taken into account for such a decision. These principles encompass patient privacy, data ownership, individual autonomy, confidentiality, necessity of data processing, non-maleficence and beneficence^1,33^. Considered jointly, they result in a trade-off to be made between the preservation of ethical rights of treated patients and the beneficence of the scientific project^15,18,26^. Criticism often arises concerning the prevailing trade-off in favor of patients’ privacy, where ethics committees tend to overprotect patient data^23,27^. What is frequently overlooked is the ethical responsibility to share and reuse medical RWD to advance medical progress in diagnoses and treatment. Thus, a consequence of overprotecting data is suboptimal patient care which is, in turn, unethical^1,9,26^. Measures to prevent overprotection are increasing the awareness of its risks through education, as well as the development of clear ethical regulations and guidelines^28^. To facilitate the latter, the data set compilation process for medical RWD should be simplified, e.g. by standardization of processes and data formats because its current complexity challenges the creation of regulations and guidelines^17^.

#### Uncertainties in project planning

Many of the mentioned concerns related to legal and ethical requirements occur during project planning and design. Here a variety of decisions are made regarding the composition of the RWD set and its processing. These affect all subsequent project steps, but must be determined at an early stage if the project framework necessitates approvals from governing entities. This is because the governing entities require all planned processing steps to be documented in a study plan, serving as the foundation for their decision-making process. This results in long project planning phases due to uncertainties in a complex multi-player environment^13–16,21^. Additionally, creating a strict study plan usually works for clinical trials, but in data science, meaningful results often require more flexibility. For instance, it might be necessary to redesign the project plan throughout data processing. Therefore, project frameworks that show researchers how to reshape their project in specific cases would be much better suited for secondary use of medical RWD^25,34^. Taking it a step further, a general guideline or regulation on how to conduct MDS projects would decrease planning time and the risk of errors, both of which are higher if each project is designed individually^14^. To already now minimize the uncertainties in project planning and, thereby, the duration of the planning phase, research teams should communicate intensely and collaboratively plan their tasks^9,18^. Since this is a challenging task in a highly interdisciplinary environment, early definition of structures, binding deadlines, and clear assignment of responsibilities, such as designating a person responsible for timely data provision in each department, are crucial^8,14^.

#### The role of the patient consent

As mentioned in the introduction to this section 3.1, dynamical consent management allowing the patients to effectively give and withdraw their consent at any point in time is a crucial measure to foster patient empowerment. As a result, it also leads to more acceptance of MDS by the affected individuals. Furthermore, in section 3.1.1 the informed patient consent was mentioned as a possible legal justification for processing personal sensitive data. However, the traditional informed consent requires patients to explicitly consent to the specific processing of their data. This means their consent is tied to a specific project^35,36^. For retrospective projects such a consent cannot be obtained during the patients’ stay at the hospital because the project idea does not exist at that time. Hence, the researcher would have to retrospectively contact all patients whose data is needed for the project, describe the project objective and methodology to them and then ask for their consent. This requires great effort, is, itself, questionable in terms of data protection and even not feasible if the patients are deceased. Making clinical data truly reusable in a research context, therefore, requires a broad consent in which the patients generally agree to the secondary use of their data in ethically approved research contexts. Furthermore, the retrieval of such a broad consent must be integrated into daily clinical routine and the consent management needs to be digitized. Otherwise, the information about the patient consent status might not be easily retrievable for the researcher^8,18,21,37^.

Previous research has documented that most patients are willing to share their data and even perceive sharing their medical data as a common duty^38^. Therefore, it is highly likely that extensively introducing a broad consent such as the one developed by the MI-I in Germany into clinical practice, combined with a fully digital and dynamic consent management, would have a significant positive impact on the feasibility of MDS projects^39^. It would allow patients to actively determine which future research projects may use their data.

### Technical challenges

**Fig. 2:**
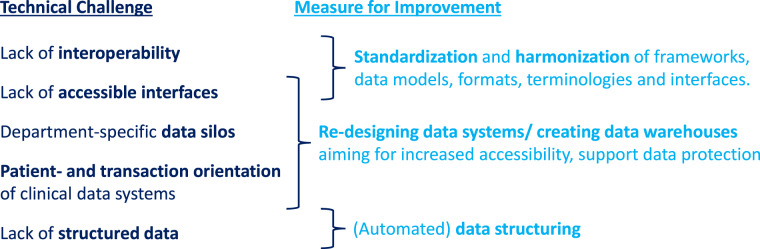
Technical challenges of curating medical RWD sets and possible measures for improvement.

When describing the challenges resulting from balancing benefits and harms in MDS projects, some measures were suggested that require technical solutions. One example for this is the implementation of data protection measures like data access control, safe data transfer, encryption, or de-identification^20^. However, there are not only technical solutions but also challenges, as shown in Fig. 2.

One category of technical challenges results from the specificities of medical data outlined in section 2. Medical RWD is characterized by a higher level of heterogeneity regarding data types and feature availability than data from any other scientific field^18,19,26^. Thus, compiling usable medical data sets from RWD requires the technical capabilities of skillful data integration, type conversion and data imputation. However, heterogeneity is not restricted to data formats. A common problem is differences in the primary purpose of data acquisition or primary care leading to different data formats and standards being used^8^. This results in different physicians, clinical departments, or clinical sites not necessarily using the same data scales or units, syntax, data models, ontology, or terminology. Hence, it is difficult to decide which standards to use in an MDS project. A subsequent challenge arising from this lack of interoperability is the conversion between standards that potentially leads to information loss^19,26,40^. Last but not least, heterogeneity is also reflected in different identifiers being used in different sites. This challenges the linkage of related medical records, which may even become impossible once the data is de-identified^41^. Promising and important measures to meet the challenges concerning heterogeneity are the development, standardization, harmonization and, eventually, deployment of conceptual frameworks, data models, formats, terminologies, and interfaces^8,13,14,16,42^. An example illustrating the feasibility and effectiveness of these measures is the widely used DICOM standard for Picture Archiving and Communications systems (PACS)^18^. Similar effects are expected from the deployment of the HL7 FHIR standard for general healthcare related data that is currently being developed^43^. However, besides appreciating the benefits of new approaches, the potential of already existing standards like the SNOMED CT terminology should not be neglected. It still has limitations, such as its complexity challenging the identification of respectively fitting codes and its incompleteness partly requiring to add own codes. On the other hand, SNOMED CT is already very comprehensive. Once its practical applicability is improved, SNOMED CT could be introduced as an obligatory standard in medical data systems fostering interoperability^13,16,42^.

Another significant technical challenge is the fact that a majority of medical RWD is typically available in a semi-structured or unstructured format, while the application of most machine learning algorithms necessitates structured data^8,19,42,44^. Primary care documentation often relies on free text fields or letters because they can capture all real-world contingencies while structured and standardized data models cannot. Additionally documenting the cases in a structured way, is too time-consuming for clinical practice. So, the primary clinical systems mainly contain semi-structured or unstructured RWD^7,13,23^. To increase the amount of available structured data, automated data structuring using Natural Language Processing (NLP) is a possible solution. However, it is not easy to implement for various reasons. Among them are the already mentioned inconsistent application of terms and abbreviations in medical texts and the requirement to manually structure some free text data sets to get annotated training data^13,42^.

Workflows in primary care settings not only lead to predominantly semi-structured or unstructured documentation of medical cases, but also greatly influence the design of clinical data management systems. In primary care and administrative contexts, such as accounting, clinical staff typically need a comprehensive overview of all data pertaining to an individual patient or case. As a result, clinical data management systems have been developed with a case- or patient-centric design that presents data in a transaction-oriented manner. However, this design is at odds with the need for query-driven extract-transform-load (ETL) processes when accessing data for MDS projects. These projects typically require only a subset of the available data features, but for a group of patients^8,26^. Developing a functional ETL pipeline is further complicated by the overall lack of accessible interfaces to the data management systems and the fragmented distribution of data across various clinical departments’ systems^8,13^.

This means the design of primary clinical systems could be improved significantly if it allowed for more flexibility, i.e. support patient- and case-centricity for primary care as well as data-centricity for secondary use. Moreover, the system design should comply with data specifications and developed standards rather than requiring the data to be created according to system specifications^13^. However, a complete redesign of primary clinical systems is most likely not feasible. An alternative solution is creating clinical data repositories in the form of data lakes or data warehouses that extract and transform medical RWD from primary systems and make it usable for research^45,46^. In this context, the use of standardized platforms and frameworks such as OMOP or i2b2 further increases the interoperability of the collected data^47^. In Germany, the MI-I established DIC and MeDIC whose goal is the creation of such data repositories for the medical RWD gathered at German university hospitals. As a common standard they agreed on the HL7 FHIR based MI-I core data set (CDS)^48^. Because this is work in progress and the data repositories are populated with data from primary clinical systems, the DIC and MeDIC still need to address the challenges identified in this comment paper to create FAIR data repositories for research.

## Can we enable practical and FAIR research on medical real-world data?

The previous section has shown that compiling medical RWD sets for research carries several cultural and technical challenges. We can see that classical medical research and data science on RWD have not yet reached agreement. At university hospitals, there is still a clear focus on primary care and traditional clinical trials that is at odds with the demands of data science. Besides the technical and regulatory conflicts, there is the conflict between the principle of data minimization in medical research contradicting the explorative big data approach of data science. Thus, it should be assessed by governing entities whether the beneficence of explorative big data outweighs the ethical benefits of data minimization.

Another important measure to enable FAIR MDS is to offer data systems, e.g. data repositories, meeting the needs of data scientists. These systems should enable comprehensive query-driven data exports and increase interoperability by using shared coding systems and terminologies. To simultaneously foster compliance to legal and ethical requirements, the systems should follow the paradigm of Privacy by Design, i.e. enforcing data protection e.g. by authorization, authentication and only allowing de-identified data to be exported. A resulting positive effect would be a decrease in uncertainties for the researchers since they would have to deal with fewer concerns about data protection and security. As long as the data infrastructure does not follow Privacy by Design, the uncertainties about the secondary use of routine clinical data remain for researchers, e.g. when determining the correct legal basis for the processing of medical RWD or designing the project aiming for ethical compliance. A possible measure to decrease these uncertainties is the simplification of project approval processes, e.g. by only requiring a single project application to be sent to an interdisciplinary deciding committee covering ethics, data security and data protection. Further simplification could be achieved by requesting flexible project frameworks rather than strict project plans from the researchers in the design phase. On the part of patients and governing entities, uncertainty regarding the justification of an MDS analysis often manifests itself in the form of overprotection. Section 3.1 described that an important measure to mitigate all such concerns is offering trainings for researchers, governing entities and patients. Moreover, enhanced patient engagement in form of open science communication and dynamic consent management could further decrease the ambivalence of patients. Secondly, a digital and dynamic consent management would increase the availability and reliability of the information whether a patient currently consents to the secondary usage of their data.

Considering FAIRness as the gold standard for scientific usability of data, the current usability level of medical RWD for MDS can be improved significantly:

- **Findability**: The data system infrastructure at university hospitals is so fragmented that most data features are only findable with intense communication or experience, either from previous projects or clinical routine. Systematic investigation on available features in the individual data systems and the creation of data repositories as carried out by the DIC and MeDIC of the MI-I could help to increase findability.
- **Accessibility**: The access to medical data is currently complicated by uncertainties regarding privacy protection, complex ethico-legal requirements and the design of primary clinical systems lacking query orientation and accessible interfaces. Redesigning the systems or creating data repositories aiming for Privacy by Design and technical accessibility of clinical data would significantly ease the compilation of medical RWD sets for research.
- **Interoperability**: The interoperability is currently mainly restricted to the usage of the same patient identifiers within a hospital. Different departments often use different documentation policies, abbreviations, units, or own case IDs while different hospitals use different patient identifiers. Standardization as an agreement on common terminology, data models and coding systems would help to increase interoperability.
- **Reusability**: Given the current legal situation, true reusability is only achievable with anonymized data sets or a broad patient consent allowing the processing of patient data in ethically approved MDS projects. Otherwise, data sets are compiled and used on a project-specific basis. Once the legal basis for creating a reusable data set is established and implemented, metadata documenting data provenance should be created to further promote reusability.

To conclude, reusing medical RWD in MDS is not infeasible, but the current situation still poses a variety of challenges. This comment paper has outlined these challenges from the research perspective with a special focus on the situation in Germany and proposed high-level measures on how to effectively address them. Implementing these measures will itself be a big challenge but significantly increase the usability of medical RWD for MDS and hence promote improvements in future healthcare. Thereby the technical changes will be easier to implement than the cultural ones.

## References

[1] Gruson D, Helleputte T, Rousseau P, Gruson D (2019). Data science, artificial intelligence, and machine learning: opportunities for laboratory medicine and the value of positive regulation. Clinical biochemistry.

[2] Fröhlich H (2018). From hype to reality: data science enabling personalized medicine. BMC medicine.

[3] Thrall JH (2018). Artificial intelligence and machine learning in radiology: opportunities, challenges, pitfalls, and criteria for success. Journal of the American College of Radiology.

[4] Boehm KM, Khosravi P, Vanguri R, Gao J, Shah SP (2022). Harnessing multimodal data integration to advance precision oncology. Nature Reviews Cancer.

[5] Behrad, F. & Abadeh, M. S. An overview of deep learning methods for multimodal medical data mining. *Expert Systems with Applications* 117006 (2022).

[6] Zakim D, Schwab M (2015). Data collection as a barrier to personalized medicine. Trends in pharmacological sciences.

[7] Khozin S, Blumenthal GM, Pazdur R (2017). Real-world data for clinical evidence generation in oncology. JNCI: Journal of the National Cancer Institute.

[8] Gehring S, Eulenfeld R (2018). German medical informatics initiative: unlocking data for research and health care. Methods of information in medicine.

[9] Krumholz HM, Terry SF, Waldstreicher J (2016). Data acquisition, curation, and use for a continuously learning health system. Jama.

[10] Wilkinson MD (2016). The fair guiding principles for scientific data management and stewardship. Scientific data.

[11] Sinaci AA (2020). From raw data to fair data: the fairification workflow for health research. Methods of information in medicine.

[12] Semler SC, Wissing F, Heyder R (2018). German medical informatics initiative. Methods of information in medicine.

[13] Haarbrandt B (2018). Highmed–an open platform approach to enhance care and research across institutional boundaries. Methods of information in medicine.

[14] Prasser F, Kohlbacher O, Mansmann U, Bauer B, Kuhn KA (2018). Data integration for future medicine (difuture). Methods of information in medicine.

[15] Winter A (2018). Smart medical information technology for healthcare (smith). Methods of information in medicine.

[16] Prokosch H-U (2018). Miracum: medical informatics in research and care in university medicine. Methods of information in medicine.

[17] Ethikrat, D. Big data und gesundheit–datensouveränität als informationelle freiheitsgestaltung. Stellungnahme, Deutscher Ethikrat. Vorabfassung (2017).

[18] He J (2019). The practical implementation of artificial intelligence technologies in medicine. Nature medicine.

[19] Lee CH, Yoon H-J (2017). Medical big data: promise and challenges. Kidney research and clinical practice.

[20] Kubben, P., Dumontier, M. & Dekker, A. *Fundamentals Of Clinical Data Science* (Springer Nature, 2019). 31314217

[21] Chen D (2019). Deep learning and alternative learning strategies for retrospective real-world clinical data. NPJ digital medicine.

[22] Newaz AI, Sikder AK, Rahman MA, Uluagac AS (2021). A survey on security and privacy issues in modern healthcare systems: Attacks and defenses. ACM Transactions on Computing for Healthcare.

[23] Köngeter, A., Jungkunz, M., Winkler, E. C., Schickhardt, C. & Mehlis, K. Sekundärnutzung klinischer daten aus der patientenversorgung für forschungszwecke–eine qualitative interviewstudie zu nutzen-und risikopotenzialen aus sicht von expertinnen und experten für den deutschen forschungskontext. In *Datenreiche Medizin und das Problem der Einwilligung*, 185–210 (Springer, Berlin, Heidelberg, 2022).

[24] Skovgaard LL, Wadmann S, Hoeyer K (2019). A review of attitudes towards the reuse of health data among people in the european union: The primacy of purpose and the common good. Health policy.

[25] Mannheimer S, Pienta A, Kirilova D, Elman C, Wutich A (2019). Qualitative data sharing: Data repositories and academic libraries as key partners in addressing challenges. American Behavioral Scientist.

[26] Meystre SM (2017). Clinical data reuse or secondary use: current status and potential future progress. Yearbook of medical informatics.

[27] Prainsack, B. & Spector, T. Ethics for healthcare data is obsessed with risk–not public benefits. *The conversation* (2018).

[28] Salerno J, Knoppers BM, Lee LM, Hlaing WM, Goodman KW (2017). Ethics, big data and computing in epidemiology and public health. Annals of Epidemiology.

[29] McLennan, S. Die ethische aufsicht über die datenwissenschaft im gesundheitswesen. In *Datenreiche Medizin und das Problem der Einwilligung*, 55–69 (Springer, Berlin, Heidelberg, 2022).

[30] Shabani M, Borry P (2018). Rules for processing genetic data for research purposes in view of the new eu general data protection regulation. European Journal of Human Genetics.

[31] Krawczak, M. & Weichert, T. Vorschlag einer modernen dateninfrastruktur für die medizinische forschung in deutschland (version 1.3). Manuskript, Netzwerk Datenschutzexpertise (2017).

[32] Weichert, T. *Datenschutzrechtliche Rahmenbedingungen Medizinischer Forschung* (Medizinisch Wissenschaftliche Verlagsgesellschaft, Berlin, 2022).

[33] Rumbold JM, Pierscionek BK (2017). A critique of the regulation of data science in healthcare research in the european union. BMC medical ethics.

[34] Natarajan, P., Frenzel, J. C. & Smaltz, D. H. *Demystifying Big Data And Machine Learning For Healthcare* (CRC Press, 2017).

[35] Vlahou A (2021). Data sharing under the general data protection regulation: time to harmonize law and research ethics?. Hypertension.

[36] Hallinan D (2020). Broad consent under the gdpr: an optimistic perspective on a bright future. Life sciences, society and policy.

[37] Sun, W. *et al*. Data processing and text mining technologies on electronic medical records: a review. *Journal of healthcare engineering* **2018** (2018). 10.1155/2018/4302425 PMC5911323 29849998

[38] Richter G, Borzikowsky C, Hoyer BF, Laudes M, Krawczak M (2021). Secondary research use of personal medical data: patient attitudes towards data donation. BMC medical ethics.

[39] Zenker S (2022). Data protection-compliant broad consent for secondary use of health care data and human biosamples for (bio) medical research: towards a new german national standard. Journal of Biomedical Informatics.

[40] Huang MZ, Gibson CJ, Terry AL (2018). Measuring electronic health record use in primary care: a scoping review. Applied clinical informatics.

[41] Stammler S (2022). Mainzelliste secureepilinker (mainsel): privacy-preserving record linkage using secure multi-party computation. Bioinformatics.

[42] Vuokko, R., Mäkelä-Bengs, P., Hyppönen, H. & Doupi, P. Secondary use of structured patient data: interim results of a systematic review. In *MIE*, 291–295 (2015). 25991152

[43] Rinaldi E, Saas J, Thun S (2021). Use of loinc and snomed ct with fhir for microbiology data. Studies in health technology and informatics.

[44] Kindermann, A. *et al*. Preliminary analysis of structured reporting in the highmed use case cardiology: challenges and measures. *Stud Health Technol Inform (Forthcoming)* (2021). 10.3233/SHTI210068 34042893

[45] Hamoud, A., Hashim, A. S. & Awadh, W. A. Clinical data warehouse: a review. *Iraqi Journal for Computers and Informatics* **44** (2018).

[46] Cappiello, C., Gribaudo, M., Plebani, P., Salnitri, M. & Tanca, L. Enabling real-world medicine with data lake federation: A research perspective. In *VLDB Workshop on Data Management and Analytics for Medicine and Healthcare*, 39–56 (Springer, 2022).

[47] Rinner, C., Gezgin, D., Wendl, C. & Gall, W. A clinical data warehouse based on omop and i2b2 for austrian health claims data. In *eHealth*, 94–99 (2018). 29726424

[48] Medical Informatics Initiative. The medical informatics initiative’s core data set. https://www.medizininformatik-initiative.de/en/medical-informatics-initiatives-core-data-set. Online; accessed 16-June-2023 (2017).

